# Beach landscape Dataset of Fernando de Noronha Island (Brazil)

**DOI:** 10.1016/j.dib.2020.105672

**Published:** 2020-05-13

**Authors:** Luana Carla Portz, Samanta da Costa Cristiano, Gabriela Camboim Rockett

**Affiliations:** a Civil and Environmental Department, Universidad de la Costa, Barranquilla, Atlántico, Colombia; b Programa de Pós-Graduação em Gerenciamento Costeiro, Universidade Federal do Rio Grande, RS, Brazil; c Universidade Federal do Rio Grande do Sul/Campus Litoral Norte, Centro de Estudos Costeiros, Limnológicos e Marinhos (CECLIMAR), Brazil

**Keywords:** Coastal scenarios, Checklist, Fuzzy logic

## Abstract

Beach landscape Dataset of Fernando de Noronha Island (Brazil), using a checklist with 26 physical and human parameters. Fernando de Noronha beaches were divided into sectors according to the landscape diversity. In total, 19 sectors were evaluated based on observations done during walks in the area, observations from viewpoints, with remote data. The evaluations were performed during fieldwork from 2014 (summer) and 2016 (spring). The landscape quality evaluation of Fernando de Noronha was performed using the Coastal Scenery Evaluation System. This method converts qualitative-quantitative data in quantitative data by estimating weights for 26 parameters (18 physical parameters – P - and 8 human-related parameters – H). The main parameters that define the landscape quality are classified from 1 (absence/bad quality) to 5 (presence/excellent quality). A mathematical model based on fuzzy logic was utilized to integrate the parameters weights in a special system for the scenarios classifications resulting in a value named D. The D-value is the indicator of the attractiveness of the evaluated place. The beaches are divided into classes ranging from 1 (extremely attractive natural site) to 5 (unattractive urban areas).

Specifications Table

**Table:** 

Subject	Nature and Landscape Conservation
Specific subject area	Coastal scenarios methodology application in Fernando de Noronha Island (Brazil)
Type of data	Table
	Graph
	Figure
How data were acquired	Field trip, GNSS, data analysis and interpretation by ArcGIS program.
Data format	Raw
	Analyzed
	KMZ
Parameters for data collection	Field observation by researchers from different fields of knowledge. Two distinct periods of the year were considered.
Description of data collection	Data collection was made in field work observations of the landscape.
Data source location	Fernando de Noronha Island, Brazil
	3° 51′ 13.71″ S, 32° 25′ 25.63″ W
Data accessibility	https://data.mendeley.com/datasets/42ndmsmszk/draft?a=6bda2b9a-f6b2-4724-823f-2e367501f683
Related research article	Samanta da Costa Cristiano, Gabriela Camboim Rockett, Luana Carla Portz, José Rodrigues de Souza Filho. Beach landscape management as a sustainable tourism resource in Fernando de Noronha Island (Brazil). Marine Pollution Bulletin 150 (2020) 110621. https://doi.org/10.1016/j.marpolbul.2019.110621

## Value of the data

• The data can be useful for coastal management in island and protected areas.
• Any researcher that deals with the Fernando de Noronha Island can benefit from these datasets. In addition, researchers from other fields of knowledge, decision makers and public officials.
• The data can be useful for further researches that deal with any coastal scenarios or coastal management research.
• Beach landscape Dataset create new perspectives for sustainable development based on the singularities of this touristic resource – the landscape.
• The data investigate extensively the effect of tourism on changes in the coastal scenery, including natural and anthropogenic parameters.

## 1 Data Description

**Fig. 1:**
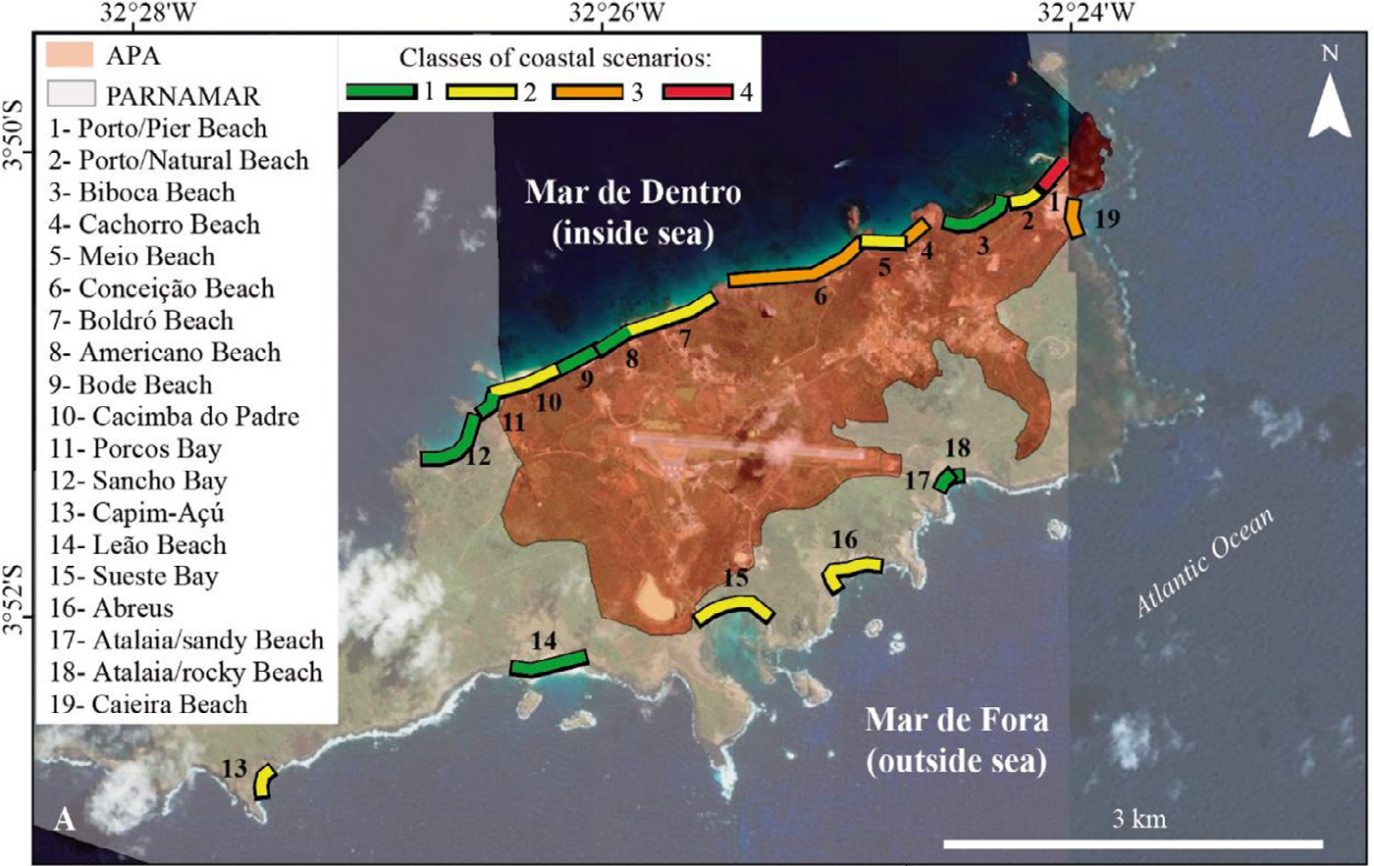
Location of the Fernando de Noronha island beaches (Brazil) analyzed and specific sectors of APA and PARNAMAR areas. (Spatial data sources: Basemap: ESRI; APA/PARNAMAR delimitation Shapefile: ICMBio).

The dataset consists in the classification of Fernando de Noronha's beaches in classes (1-4), according to the Coastal Scenarios. The classification is in Fig. 1. The highest diversity of classes is in the APA/Mar de Dentro area (Fernando de Noronha Environmental Protection Area-APA). On the other hand, in the PARNAMAR/Mar de Fora area (Fernando de Noronha National Marine Park-PARNAMAR), there is only one beach in class 3 (mostly natural areas, with some landscape parameters that stands out), and all the other beaches are in classes 1 (extremely attractive natural sites) and 2 (natural, attractive areas with high landscape value sites) (Fig. 1 and Table 1).

**Tabla 1:** Summary of the results, average D-value (classes) (spring and summer).

ID Beach/Bay		Sector	D-values	D-values summer	Class summer	D-values spring	Class spring
1	Porto/Pier Beach	APA	0.35	0,36	4	0.35	4
2	Porto/natural Beach	APA	0.77	0,76	2	0.78	2
3	Biboca Beach	APA	1.09	1,09	1	1.09	1
4	Cachorro Beach	APA	0.43	0.50	3	0.37	4
5	Meio Beach	APA	0.76	0.75	2	0.78	2
6	Conceição Beach	APA	0.63	0.57	3	0.70	2
7	Boldró Beach	APA	0.77	0.76	2	0.79	2
8	Americano Beach	APA	1.12	1.12	1	1.13	1
9	Bode Beach	APA	1.01	0.98	1	1.04	1
10	Cacimba do Padre	APA	0.84	0.79	2	0.89	1
11	Porcos Bay	PARNA	1.03	1.02	1	1.05	1
12	Sancho Bay	PARNA	1.20	1.20	1	1.20	1
13	Capim-Açú	PARNA	0.74	0.74	2	0.74	2
14	Leão Beach	PARNA	1.15	1.15	1	1.15	1
15	Sueste Bay	PARNA	0.71	0.71	2	0.71	2
16	Abreus	PARNA	0.79	0.79	2	0.79	2
17	Atalaia/sand Beach	PARNA	1.10	1.10	1	1.10	1
18	Atalaia/rocky Beach	PARNA	0.99	0.99	1	0.99	1
19	Caieira Beach	PARNA	0.47	0.48	3	0.46	3

The D value (attractiveness indicator) is presented for summer and spring periods (Table 1).

**Fig. 2:**
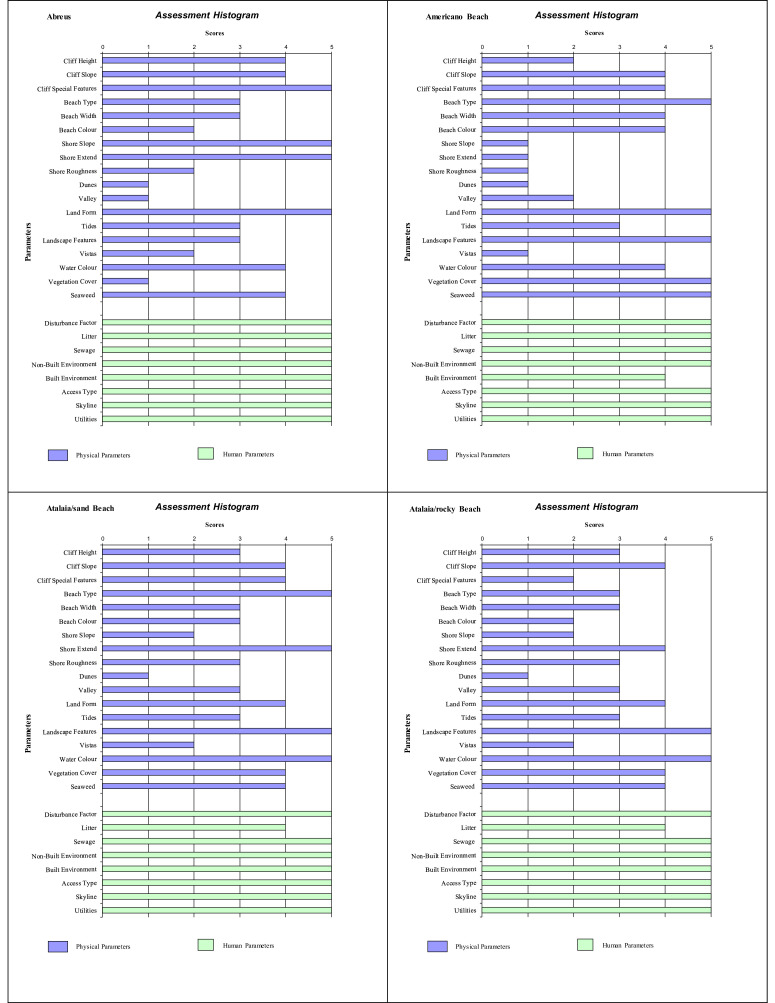

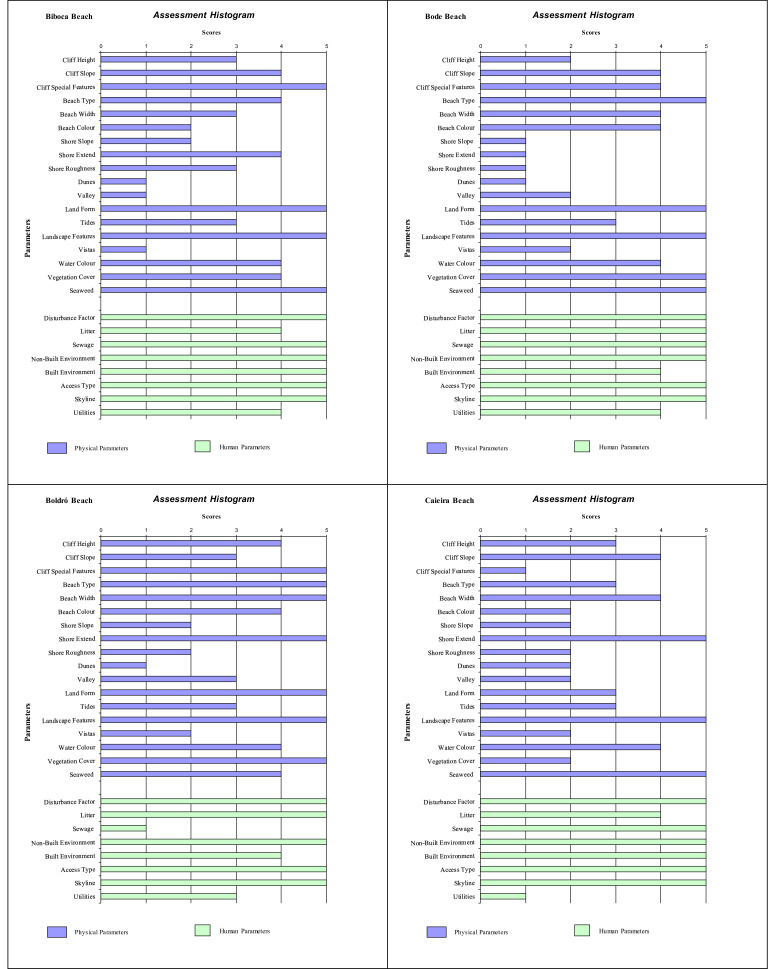

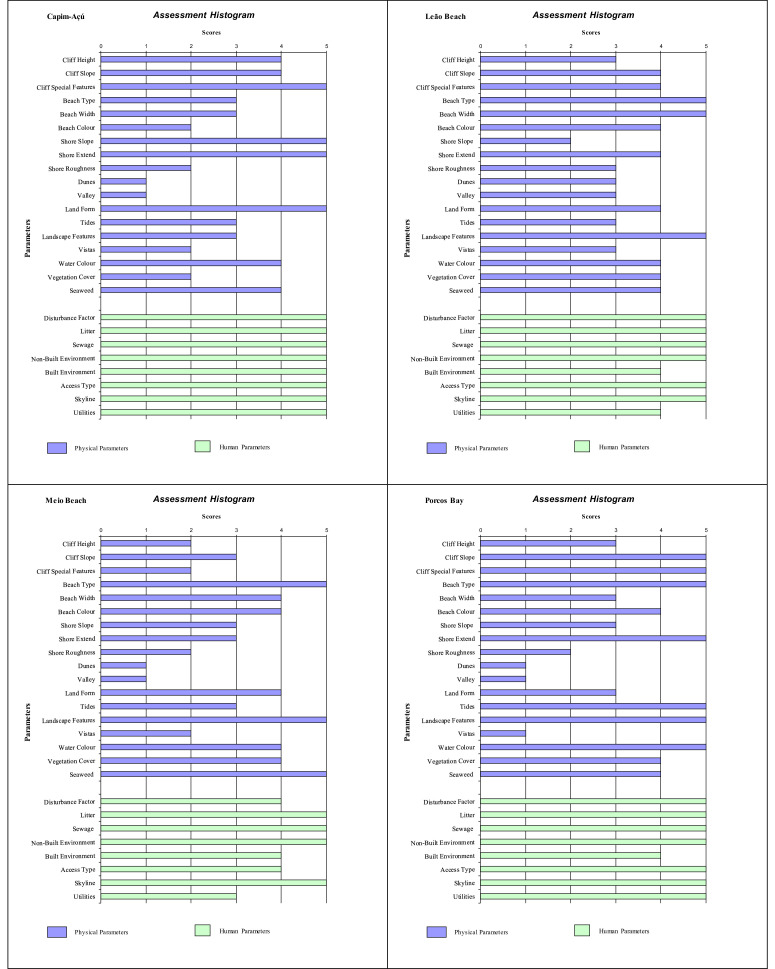

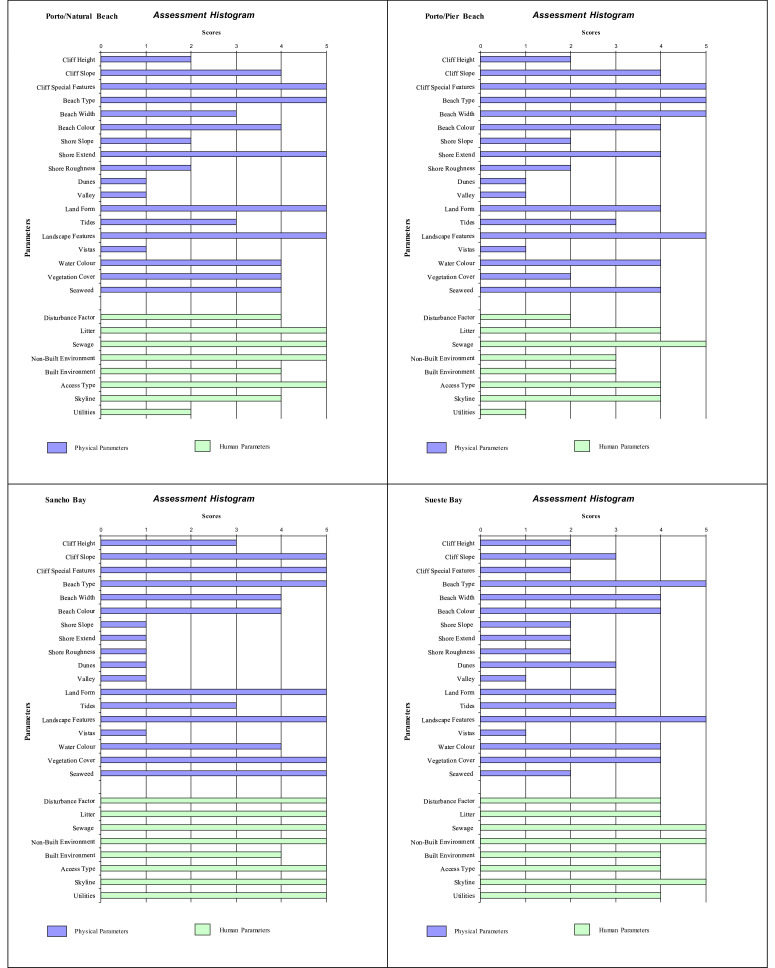
Histogram with variations in the attributes’ values of categories 1 to 5 (to see these categories look at Table 1).

**Fig. 3:**
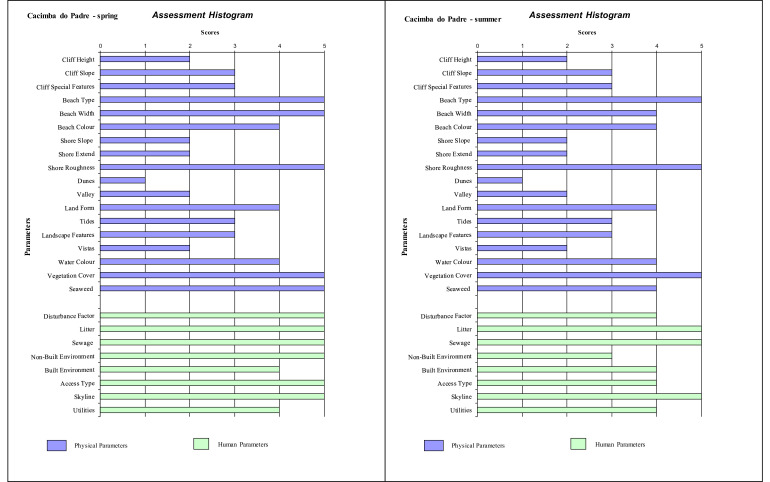
Histogram with variations in the attribute's values of categories 1 to 5 (to see these categories look at Table 1) for Cacimba do Padre Beach (Spring and Summer).

**Fig. 4:**
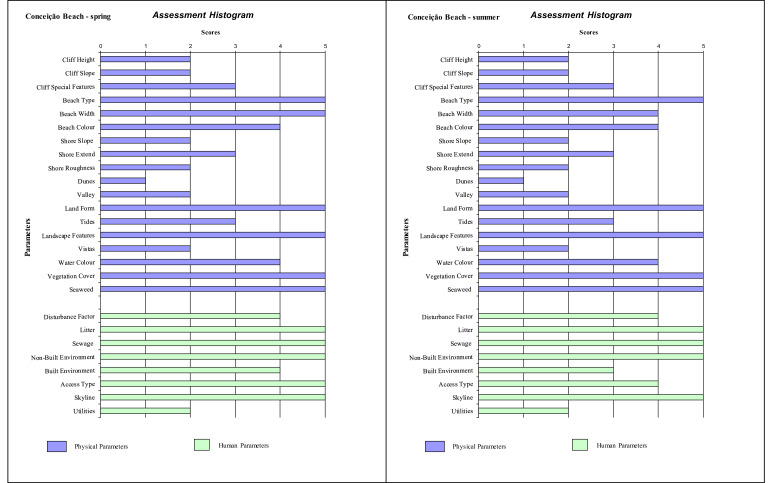
Histogram with variations in the attribute's values of categories 1 to 5 (to see these categories look at Table 1) for Conceição Beach (Spring and Summer).

**Fig. 5:**
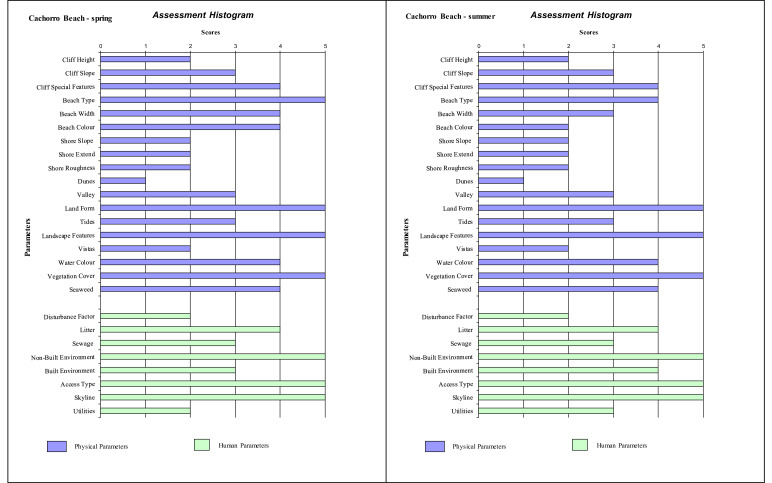
Histogram with variations in the attribute's values of categories 1 to 5 (to see these categories look at Table 1) for Cachorro Beach (Spring and Summer).

Histograms provide a visual summary of the physical and human parameters obtained through the application of the checklist and are useful for immediate evaluation of high and low ranking attributes. The values defined for each parameter (physical and human) are shown in Fig. 2 (beaches without seasonal variations). Seasonal changes in the parameters can be seen in the histogram (Figs. 3, Fig. 4, Fig. 5). As an example, Cacimba do Padre Beach changes from class 1 in the spring season (D-value = 0.89) to class 2 in the summer season (D-value = 0.79) (Table 1) (Fig. 3).

Attribute values 4 or 5 produces a high scenic value (high rating). The predominance of values 4 and 5 in phisical and anthropogenic parameters in the histogram can be observed for Leão Beach and Sancho Bay (Fig. 3). On the other hand, attribute values 1 or 2 produces a high scenic value (high rating), indicating the unfavorable impact on physical and / or human parameters. In this assessment, it is generally the human parameters that reduce the assessment, such as Porto/Pier Beach (Fig. 2).

## 2 Experimental Design, Materials, and Methods

The methodology used to perform the beach landscape quality evaluation of Fernando de Noronha island beaches (Brazil) was the Coastal Scenery Evaluation System [1], [2], [3]. This method consists of estimating weights for 26 parameters (18 physical parameters – P - and 8 human-related parameters – H) and converting qualitative-quantitative data in quantitative data. According to the Evaluation System, the 26 parameters were considered essential for an attractive coastal landscape and are shown in Table 1. The parameters are weighted from 1 to 5, where: 1 refer to the item's “absence or bad quality” and 5 refers to the item's “presence or excellent quality”.

For the evaluation of Fernando de Noronha island beaches, the beaches were divided in 19 homogeneous landscape sectors, (shown in Fig. 1). Some beaches, due to its and landscape variation (heterogeneity), were divided into more than one sector (e.g. Atalaia/rocky beach and Atalaia/sandy beach; Porto/Pier Beach and Porto/Natural Beach). From the total beach sectors, 10 of them are inside the APA area and 9 of them are inside the PARNAMAR area (Fig. 1).

The evaluation of each beach sector and checklist filling was performed (i) in fieldwork - using landscape observations during walks in the beaches and/or from viewpoints, and (ii) in data remote-check using Google Earth imagery. Due to seasonal variability, landscape evaluations were performed during spring and during summer - summer fieldwork performed in 2014 and spring fieldwork performed in 2016. The professionals involved in the field evaluations were from the areas of biosciences and geosciences/geography.

After each parameter's evaluation (checklist), data processing was performed, in order to integrate the parameters weights in a special system for the scenarios classifications. A graphical summary of the investigated sceneries were obtained/generated from the weighted averages and association degrees and histograms [4]. Beach scenery is better when most of the parameters scores “5” (which result in a right-leaning association degree curve), and in the same way the potential status of the scenic assessment are indicated from the weighted average – the more parameters scoring “5”, the better the coastal scenery. For this integration, a mathematical model based on fuzzy logic is used and the result obtained from this model is a value named D (D-value), which is the indicator of the attractiveness of the evaluated beach. According to the Method [1], there are five possible beach classes, according to the D-value obtained (Table 2).

**Table 2:** Classification of Beach landscape quality, according to the D-value obtained from the Coastal Scenery Evaluation System [1].

D-Value	Beach Landscape Quality Class	Class Description
D >0.85	1	extremely attractive natural site;
0.85> D ≥ 0.65	2	natural, attractive areas with high landscape value sites;
0.65> D ≥ 0.4	3	mostly natural areas with some landscape values highlighted;
0.4> D ≥0	4	urban areas, mainly unattractive, with few landscapes’ values highlighted;
D <0	5	unattractive urban areas, with intense development and low landscape values.

Class 1 beaches are extremely attractive natural sites, with a D-value >0.85. Class 2 beaches are natural, attractive areas with high landscaping value site and a D-value between 0.65 and 085. Class 3 beaches are mostly natural areas with some landscaping value highlighted, and D-value between 0.65 and 0.40. Class 4 beaches are urban areas, mainly unattractive, with few landscaping values highlighted, and D-value between zero and 0.40. Class 5 beaches are unattractive urban areas, with intense development and low landscaping value, and D-value below zero.

The data obtained in this study are available as georreferenced files (.kmz)

## CRediT authorship contribution statement

**Luana Carla Portz:** Conceptualization, Formal analysis, Investigation, Methodology, Visualization, Writing - original draft, Writing - review & editing. **Samanta da Costa Cristiano:** Conceptualization, Data curation, Formal analysis, Investigation, Methodology, Resources, Visualization, Writing - original draft, Writing - review & editing. **Gabriela Camboim Rockett:** Conceptualization, Data curation, Formal analysis, Investigation, Methodology, Resources, Visualization, Writing - original draft, Writing - review & editing.
